# Rapid and Comprehensive Evaluation of (Poly)phenolic Compounds in Pomegranate (*Punica granatum* L.) Juice by UHPLC-MS^n^

**DOI:** 10.3390/molecules171214821

**Published:** 2012-12-13

**Authors:** Pedro Mena, Luca Calani, Chiara Dall’Asta, Gianni Galaverna, Cristina García-Viguera, Renato Bruni, Alan Crozier, Daniele Del Rio

**Affiliations:** 1 Phytochemistry Laboratory, Department of Food Science and Technology, CEBAS-CSIC, Murcia 30100, Spain; Email: pmena@cebas.csic.es (P.M.); cgviguera@cebas.csic.es (C.G.-V.); 2 The φ^2^ Laboratory of Phytochemicals in Physiology, Department of Food Science, University of Parma, Parma 43125, Italy; Email: luca.calani@unipr.it (L.C.); 3 Department of Food Science, University of Parma, Parma 43125, Italy; Email: chiara.dallasta@unipr.it (C.D.); gianni.galaverna@unipr.it (G.G.); renato.bruni@unipr.it (R.B.); 4 North Lab., School of Medicine, College of Medical, Veterinary and Life Sciences, University of Glasgow, Glasgow G12 8QQ, UK; Email: alan.crozier@glasgow.ac.uk

**Keywords:** *Punica granatum* L., source ionization conditions, ellagitannins, C18 and PFP columns, phytochemicals identification

## Abstract

The comprehensive identification of phenolic compounds in food and beverages is a crucial starting point for assessing their biological, nutritional, and technological properties. Pomegranate (*Punica granatum* L.) has been described as a rich source of (poly)phenolic components, with a broad array of different structures (phenolic acids, flavonoids, and hydrolyzable tannins) and a quick, high throughput, and accurate screening of its complete profile is still lacking. In the present work, a method for UHPLC separation and linear ion trap mass spectrometric (MS^n^) characterization of pomegranate juice phenolic fraction was optimized by comparing several different analytical conditions. The best solutions for phenolic acids, anthocyanins, flavonoids, and ellagitannins have been delineated and more than 70 compounds have been identified and fully characterized in less than one hour total analysis time. Twenty-one compounds were tentatively detected for the first time in pomegranate juice. The proposed fingerprinting approach could be easily translated to other plant derived food extracts and beverages containing a wide array of phytochemical compounds.

## 1. Introduction

Comprehensive identification of phenolic compounds in food matrices is a crucial starting point for assessing their biological, nutritional, and technological properties [1]. However, due to the complexity of plant secondary metabolism, the full characterization of phytochemicals in fruits and vegetables can be difficult, time consuming, and require sensitive and accurate techniques [2].

Pomegranate (*Punica granatum* L.) has been described as a rich source of phenolics with a plethora of health features and also playing a major role in juice quality and sensorial performance [3]. However, the broad array of different (poly)phenolic structures (phenolic acids, flavonoids, and hydrolyzable tannins) [4,5,6,7] may make the accurate screening of their profile particularly difficult. High performance liquid chromatography coupled with photodiode array detector (HPLC-DAD) has typically been the technique of choice for simultaneous identification and quantification of pomegranate phenolics [6,7,8,9,10], but during this last decade, mass spectrometric detection (MS) has also been used for the qualitative analysis and characterization of pomegranate products [11,12,13,14]. Nevertheless, the range of chemical targets was restricted to particular phenolic groups and, hence, did not account for the real phytochemical potential of pomegranate. To our knowledge, the most accurate approach carried out in order to assess the phenolic composition of pomegranate allowed the detection of 48 compounds belonging to several different (poly)phenolic groups [5]. However, the methodology proposed consisted of a hard long sample preparation and time-consuming chromatographic conditions (more than 3 h). These facts, besides a sample extraction that could alter chemical structures [15], make difficult its actual translation to routine analysis, in particular when large batches of samples have to be evaluated in a limited time.

Consequently, new approaches allowing easy sample preparation and fast chromatographic procedures for rapid screening and high throughput should be developed [16] in order to face the increasing interest for the (poly)phenolic fingerprinting of pomegranate for both quality control, germplasm screening and bioactivity evaluation purposes. Therefore, the aim of the present work was to identify the best analytical conditions needed to reach a high throughput and accurate phenolic fingerprinting of pomegranate juice by ultra-high performance liquid chromatography (UHPLC)-linear ion trap mass spectrometry. The recourse to UHPLC-MSn was selected as it might largely improve separation efficiency and method sensitivity, speeding up at the same time the chromatographic analysis [17]. Moreover, due to the different characteristics and high complexity of pomegranate phenolic compounds, single MS may hinder the screening and identification of non-target (poly)phenolic derivatives [18]. Particular care has been given to the set up of a sample preparation protocol meeting the needs for maintaining the compounds in their native form, avoiding the variability associated to processing conditions and speed up the overall process [15].

## 2. Results and Discussion

The phytochemical fingerprint of pomegranate juice was determined using a UHPLC-ESI-linear ion trap MS operating in three complementary conditions. The use of UHPLC and minimal sample preparation accounted for the feasibility of this new accurate and rapid procedure for the screening of pomegranate juices. Two experiments were performed in negative and one in positive ionization mode. The two negative ionization experiments were chosen after optimization trials focused on covering the different ionization and fragmentation capacities of the (poly)phenolic structures of pomegranate juice. Epicatechin and punicalagin were used as representative compounds for flavonoids and hydrolyzable tannins, respectively. The experiment performed in positive mode was focused on the anthocyanins profile. Analyses were carried out by using a full scan and MS^2^/MS^3^ data-dependent operative mode.

The combination of these three experiments allowed the tentative identification of a total of 75 compounds. Hydrolyzable tannins were the main class of (poly)phenolics identified in pomegranate juices. A broad number of anthocyanins, non-coloured flavonoids and phenolic acids were also found. Other phytochemicals, such as lignans, were also observed. Likewise, several organic acids were detected. The role of different analytical columns on the assessment of pomegranate juice mass profiling was also evidenced.

### 2.1. Identification of Phytochemicals Compounds in Pomegranate Juice by Complementary Analytical Conditions

The 75 compounds were identified by the interpretation of their fragmentation patterns obtained from mass spectra (MS^2^ and MS^3^ experiments). Data provided by reference standards and literature information was also employed for the comprehensive evaluation of samples. MS fragmentation patterns interpretation present in the literature was not discussed unless of special interest. The retention times and mass spectrum data along with peak assignments for compounds identified using negative ionization are described in Table 1.

#### 2.1.1. Ellagitannins

**Figure 1:**
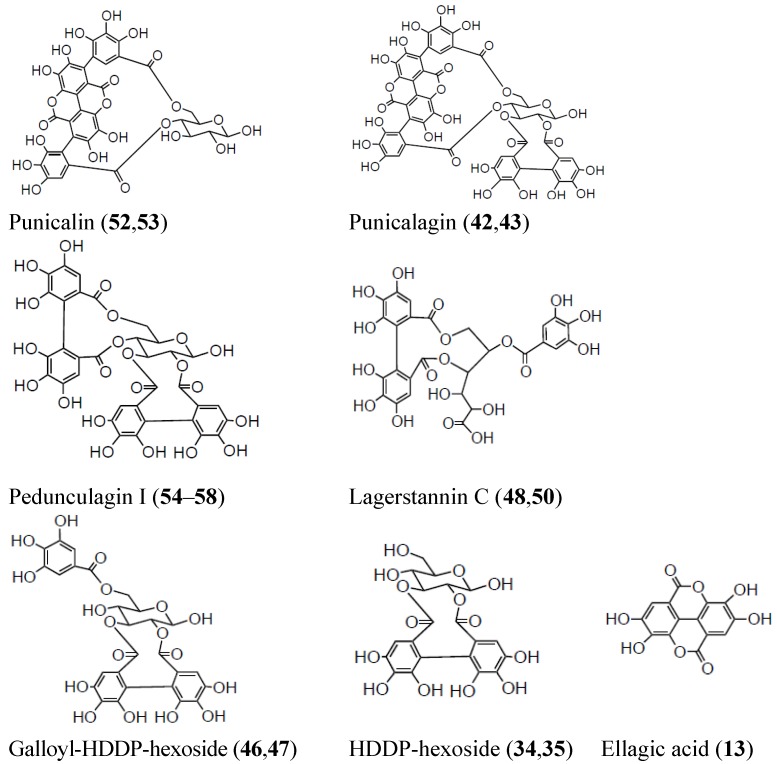
Chemical structures of hydrolyzable tannins identified in pomegranate juice.

Ellagitannins, polymeric structures including, but not limited to, different numbers of galloyl and hexahydroxydiphenoyl (HHDP) units esterified with glucose, are supposed to be the main putatively bioactive phytochemicals of pomegranate juice (Figure 1), owing to their ability to yield urolithins after metabolism within the digestive tract [19].

Up to 37 ellagitannins were detected in the pomegranate juice assessed. They were distinguished by their characteristic fragment ion spectra yielding sequential losses of galloyl (*m/z* 152), gallate (*m/z* 170), and HHDP residues (*m/z* 301) [20]. A total of 28 ellagitannins already described as present in pomegranate juice (compounds **13**, **26**, **28**, **32**, **34**, **35**, **42**, **43**, **45**–**76**) were identified by comparing with reported data [5,7,11,14,21]. Compound **68** displayed identical molecular ion (*m/z* 951) to granatin B (compound **69**). However, they differed in their MS^2^ and MS^3^ fragment ion spectra.

**Table 1:** Identification of phytochemical compounds by UHPLC-MS^n^ in negative mode under different MS operating conditions and columns.

Id.	Compounds	[M−H]^−^ (*m/z*)	MS^2^ ion fragments (*m/z*) *^c^*	MS^3^ ion fragments (*m/z*) *^c^*	BlueOrchid C18			Hypersil Gold C18			Kinetex PFP		
					RT (min)	Exp. 1	Exp. 2	RT (min)	Exp. 1	Exp. 2	RT (min)	Exp. 1	Exp. 2
**1**	L-Malic acid	**133**	**115** *^d^*, 87	71	0.84	x		0.84	x		0.79	x	
**2**	Vanillic acid *^b^*	**167**	**123**, 125, 152		8.80		x	8.52		x	9.22		x
**3**	Gallic acid *^a^*	**169**	**125**	125	2.91		x				4.78		x
**4**	Syringaldehyde *^b^*	**181**	**166**	151				8.66		x			
**5**	Citric acid	**191**	**111**, 173	111, 67	1.14	x	x	1.30	x	x	1.05	x	x
**6**	Pinocembrin *^b^*	**255**	**213**, 211, 151	213, 211, 151, 187, 169	11.69		x						
**7**	Tryhydroxyflavone *^b^*	**269**	**269**, 225, 241, 147					7.26		x			
**8**	Naringenin-like *^b^*	**271**	**151**, 177, 165, 107, 125	107, 151, 83, 65	9.20	x	x				9.04	x	x
**9**	Phloretin *^b^*	**273**	**167**	123, 125, 151	10.55		x						
**10**	(+)-Catechin *^a^*	**289**	**245**, 205, 179, 261	203, 227, 187, 161, 217	6.80	x		6.89	x		6.83	x	
**11**	(–)-Epicatechin *^a^*	**289**	**245**, 205, 179, 261	203, 227, 187, 161, 217	7.20	x	x	7.30	x	x	7.14	x	x
**12**	Hydroxybenzoic acid hexoside *^b^*	**299**	**239**, 179, 137, 209, 93	179, 137, 93							6.01	x	
**13**	Ellagic acid *^a^*	**301**	**301**, 257, 229, 185	257, 229, 301, 284, 185	8.18	x	x	8.04	x	x	8.18		x
**14**	(+)-Gallocatechin *^a^*	**305**	**179**, 221, 219, 261	164, 151, 137	6.08	x							
**15**	Vanillic acid-hexoside	**329**	**167**	123, 152, 108							4.93	x	x
**16**	Galloyl-hexoside	**331**	**169**, 241, 125, 223	125, 169	1.60	x		1.60	x				
**17**	Galloyl-hexoside	**331**	**169**, 271, 211, 193, 241	125, 169	1.79	x	x	1.79	x	x	1.79	x	x
**18**	Galloyl-hexoside	**331**	**169**, 193, 211, 271, 313	125, 169	1.96	x					1.96	x	x
**19**	Galloyl-hexoside	**331**	**271**, 169	211, 169	2.25	x	x	2.25	x	x	2.25	x	
**20**	Galloyl-hexoside	**331**	**241**, 271, 169, 313	169	3.02	x							
**21**	Galloyl-hexoside	**331**	**271**, 169, 241	211, 169	3.25	x		3.25	x	x	3.25	x	
**22**	Pinoresinol	**357**	**151**, 136, 311, 327	136, 151	8.78	x							
**23**	Secoisolariciresinol	**361**	**346**, 165, 179, 313	165, 179, 223, 122, 315	7.80		x						
**24**	Citric acid derivative	**391**	**217**, 191, 373	111, 155	1.00	x		1.40	x		1.58	x	
**25**	Coumaric acid derivative *^b^*	**429**	**163**, 265, 235, 325, 307	145, 119, 103, 89, 127	7.80	x		8.16	x		7.31	x	
**26**	Ellagic acid-pentoside	**433**	**301**	300, 257, 229	7.93	x		7.72	x	x			
**27**	Phloretin-hexoside (Phlorizin) *^a,b^*	**435**	**273**, 297	167	10.38	x							
**28**	Ellagic acid-deoxyhexoside	**447**	**300**	300, 257, 229	7.99	x	x	7.81	x	x	7.77	x	x
**29**	Kaempferol-hexoside	**447**	**285**, 284, 327, 255	257, 267, 229, 241	9.10	x							
**30**	Datiscetin-hexoside *^b^*	**447**	**285**	241, 257, 125, 217, 243	7.01		x	6.85	x	x	6.86		x
**31**	Dihydrokaempferol-hexoside	**449**	**287**, 269, 259, 431	259, 243, 269	7.32	x	x	7.49	x	x	7.18	x	x
**32**	Ellagic acid-hexoside	**463**	**301**	301, 257, 229	7.22	x	x	7.14	x	x	7.10	x	x
**33**	Quercetin-hexoside	**463**	**301**	179, 151, 257, 301, 273	8.46	x							
**34**	HHDP-hexoside	**481**	**301**, 275	301, 257, 229	1.06	x	x	1.21	x	x	1.06	x	x
**35**	HHDP-hexoside	**481**	**301**, 275	301, 257, 229	1.37	x	x	1.51	x	x	1.37	x	x
**36**	Digalloyl-hexoside	**483**	**331**, 313, 169	193, 169, 271, 211, 313	2.85	x							
**37**	Syringetin hexoside	**507**	**327**, 345, 315	312, 283	8.65	x		8.20	x		8.00	x	
**38**	Feruloyl coniferin *^b^*	**517**	**337**	193, 175, 217, 277	8.05	x							
**39**	Cyclolariciresinol hexoside *^b^*	**521**	**359**	344	10.17	x	x	8.16	x	x			
**40**	Secoisolariciresinol hexoside *^b^*	**523**	**361**, 347	346, 165, 179, 313	8.62	x		8.73		x			
**41**	Guaiacyl(8-5)ferulic acid hexoside *^b^*	**533**	**353**, 473, 443, 425, 371	338, 353, 413, 395, 371	9.24	x							
**42**	Punicalagin isomers *^a^*	**541**	**301**, 781, 601, 532, 275	301, 257, 229	6.42	x		6.42	x		6.42	x	
**43**	Punicalagin isomers *^a^*	**541**	**301**, 781, 601, 532, 275	301, 257, 229	6.75	x	x	6.75	x		6.75	x	
**44**	Kaempferol rutinoside	**593**	**285**, 547	257, 267, 241	8.90	x							
**45**	Dehydro-galloyl-HHDP-hexoside	**615**	**463**, 301, 257, 229	301	7.76	x							
**46**	Galloyl-HHDP-hexoside	**633**	**301**, 463, 275, 481	301, 257, 229	4.59	x	x	4.11	x				
**47**	Galloyl-HHDP-hexoside	**633**	**301**, 463, 275, 481	301, 257, 229	7.08	x	x	7.22	x	x			
**48**	Galloyl-HHDP-gluconate (lagerstannin C) isomer	**649**	**301**, 497	301, 257, 229	1.70	x		1.96	x		2.30	x	
**49**	Trisgalloyl glucose *^b^*	**649**	**605**, 479, 301	481, 299, 301, 425	4.29	x							
**50**	Galloyl-HHDP-gluconate (lagerstannin C) isomer	**649**	**497**, 301	301	5.78	x	x	5.90	x		6.06	x	
**51**	di(HHDP-galloylglucose)-pentose *^b^*	**707**	**783**, 613, 633, 1113, 933	481, 301, 765, 721, 275	6.22	x		6.68	x		6.25	x	
**52**	Punicalin α/A	**781**	**601**, 721	299, 271	3.66	x	x	2.59	x	x	3.10	x	x
**53**	Punicalin β/B	**781**	**601**, 721	299, 271	3.90	x	x	2.80	x	x	3.30	x	x
**54**	Pedunculagin I isomer	**783**	**481**, 721, 765, 301	437, 419, 299, 275	3.48	x	x	3.53	x	x	5.64	x	x
**55**	Pedunculagin I isomer	**783**	**301**, 481, 765	301, 257, 229	5.57	x	x	5.16	x	x	5.57		x
**56**	Pedunculagin I isomer	**783**	**301**, 481, 765	301, 257, 229	5.94	x	x	5.69	x	x	6.00	x	
**57**	Pedunculagin I isomer	**783**	**301**, 481, 765	301, 257, 229	6.53	x	x	6.53		x			
**58**	Pedunculagin I isomer	**783**	**301**, 481, 275, 765	301, 257, 229	6.65	x	x	6.65		x	6.55	x	x
**70**	Punicalagin isomer	**1083**	**1065**, 807, 601, 1021, 721	721, 575, 1047, 1021, 601	6.08	x	x	5.40	x	x	6.11		x
**42**	Punicalagin α *^a^*	**1083**	**781**, 601	601, 721	6.45	x	x				6.49	x	x
**43**	Punicalagin β *^a^*	**1083**	**781**, 601	601, 721	6.75	x	x				6.71	x	x
**71**	Digalloyl-gallagyl-hexoside	**1085**	**765**, 783, 633, 451	597, 613, 301	9.56	x	x						
**72**	Punicalagin-like	**1101**	**1083**, 781, 601	781, 601, 575, 721	2.56		x	2.56	x	x			
**51**	Di(HHDP-galloylglucose)-pentose *^b^*	**1415**	**1397**, 783, 933, 1113, 633	765, 907, 631, 1121, 1077	6.28		x	6.66		x	6.30		x
**73**	Digalloyl triHHDP-diglucose (sanguiin H10) isomer *^b^*	**1567**	**765**, 935, 783, 915, 1209	597, 401, 301, 613, 533	6.75		x						

*^a^* Compounds identified by comparing retention times and MS data with those of reference compounds; *^b^* compounds (tentatively) identified for the first time in pomegranate juice; *^c^* fragment ions are listed in order of relative abundances; *^d^* MS^2^ ions in bold were those subjected to MS^3^ fragmentation; *^e^* Exp. 1, experimental conditions 1, Exp. 2, experimental conditions 2. Compounds **42**, **43** and **51** appear as both mono- and doubly-charged molecular ions.

Compound **68** fragmented to *m/z* 907 (loss of CO_2_), 783 (loss of galloyl group), and 481 (HHDP-glu). This fragmentation pattern was similar to trisgalloyl HHDP glucose, as reported by Kähkönen *et al*. [22]. Similarly, compounds **48–50** exhibited the same molecular ion at *m/z* 649. Compound **48** and **50** were identified as lagerstannin C isomers by the characteristic fragment at *m/z* 497 as a consequence of releasing HHDP-gluconic acid. On the other hand, compound **49** released fragment ions at *m/z* 605 (loss of CO_2_), 479 (loss of galloyl group), and 481 (HHDP-glu). According to this fragmentation, compound **49** was tentatively identified as trigalloyl-glucoside [23].

**Figure 2:**
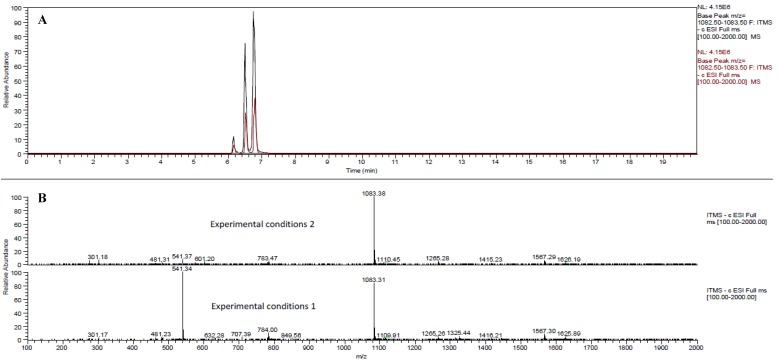
(**A**) Chromatograms of punicalagin isomer (**70**), α-punicalagin (**42**) and β-punicalagin (**43**) in Full MS scan (*m/z* 1083) for pomegranate juice analyzed by BlueOrchid C18 column. Comparison of the two different analytical conditions: experimental conditions 1 (brown peaks) and experimental conditions 2 (black peaks). (**B**) MS spectra of α- and β-punicalagin using the two experimental conditions.

Both MS operating modes (Experiments 1 and 2) displayed high performance for the identification of these polymeric structures. Actually, two thirds of the identified ellagitannins were identified under both experimental conditions. Contrary to this, the presence of one third of the whole set of ellagitannins was strictly method-dependent. This observation revealed remarkable differences in specific responses between the different ionization conditions applied. This is in good agreement with the literature, as the ionization conditions have been described as crucial for the suitable assessment of phenolic structures [24]. In the peculiar case of hydrolyzable tannins, the formation of doubly charged molecular ions has been reported when electrospray ionization was used [25]. These doubly charged ions ([M−2H]^2–^) usually generate larger fragment ions (MS^2^ and MS^3^) with respect to those found in full MS [20]. In this set of experiments, however, despite many tannins could have exhibited double charged ions, their presence was evident only for five compounds (**42**, **43**, **51**, **59** and **60**), consistently in both negative ionization experiments. Isomers **42** and **43** yielded two ions at *m/z* 1083 and 541, corresponding to singly and doubly-charged punicalagin isomer ions, respectively. The single charge *m/z* 1083 ion was detected in both experimental conditions and was characteristic of punicalagins, the main ellagitannins described in pomegranate juices [7]. The doubly charged ion, [M−2H]^2–^ 541, was mainly detected in Experiment 1 and gave MS^2^ fragments at *m/z* 301 (HHDP), 781 (gallagyl-glucose), and 601 (gallagyl), a fragmentation pattern also consistent with punicalagin. The coelution of both molecular ions confirmed this identification. Finally, this observation was confirmed by direct infusion of a punicalagin standard for both MS operating conditions, which highlighted a major abundance of *m/z* 541 over *m/z* 1083 in experiment 1, whilst *m/z* 1083 was more abundant than its double charge counterpart in experiment 2. The predominance of a molecular ion, either the mono-charged *m/z* 1083 or the doubly-charged *m/z* 541, in a determined ionization mode is shown in Figure 2.

Compound **51** displayed a [M−2H]^2–^ at *m/z* 707 (detected only for Experiment 1) and a [M−H]^–^ at *m/z* 1415 (detected only for Experiment 2). Both pseudomolecular ions showed the same MS^2^ and MS^3^ fragment ions, which were in accordance with those reported for tentative identification of a di(HHDP-galloylglucose)-pentose derivative in strawberries [20]. In addition, the fact that each molecular ion, either mono- or doubly-charged, was exclusively detected for an operating condition, emphasized once again the role of different ionization tunes in the evaluation of phytochemical profiles. However, due to the intrinsic limitations of MS methodolgies, further NMR analysis should be performed in order to confirm the presence of di(HHDP-galloylglucose)-pentose in pomegranate juice.

Compounds **59** and **60** gave a similar doubly charged molecular ion at *m/z* 783 for both experiments and fragment ion spectra characteristic of sanguiin H-10. Consequently, these compounds could be tentatively identified as sanguiin H-10 isomers, but the alternative identification of another digalloyl-triHHDP-diglucose structures (with [M−H]^–^ at *m/z* 1567) [20] could not be ruled out. A point worth mentioning is that other compounds also showed molecular ions at *m/z* 783 (compounds **54**–**58**, and **61**), although molecular ions higher than *m/z* 783 were not present in their ion spectra. Therefore, compounds **54**–**58** with [M−H]^–^ at *m/z* 783 were identified as pedunculagin I (bis-HHDP-hexoside) isomers [5], differently from the previously described compounds **59** and **60**, both exhibiting greater fragment ions (MS^2^ and MS^3^). Compound **61**, also sharing a [M−H]^–^ at *m/z* 783, was tentatively identified as casuariin, according to its fragmentation pattern [26]. Moreover, the *m/z* 1567 was not present in these tentative sanguiin H-10 isomers (compounds **59** and **60**), implying that these polymeric structures were not stable under the two ion source conditions used [27]. However, a compound (**73**) with [M−H]^–^ at *m/z* 1567 was observed in experiment 2 conditions and tentatively identified as another sanguiin H-10 isomer. In this case, the structural configuration of compound **73** may be different to that showed by compounds **59** and **60**, as fragmentation pattern was diverse. In fact, compounds **59**, **60**, and **73** could be those three stereoisomers of sanguiin H-10 found in blackberry by Gasperotti *et al*. [1].

Overall, the prevalent presence of doubly-charged ellagitannins in experiment 1, with respect to experiment 2, could be linked to differences in cone voltage and source temperature. Higher cone voltage (capillary voltage, in our equipment) leads to fragmentation of oligomers in the ESI source [28].

On the other hand, an increase in source temperature can enhance the ionization yield by reducing the heat-induced fragmentation [27]. In Experiment 2, higher source temperature and capillary voltage probably quenched the dissociation of ellagitannins in the ESI source. Therefore, fragmentation of polimeric ellagitannins at their weakest bond, the central diaryl ether function [29], may be facilitated by the complementary participation of different source conditions.

#### 2.1.2. Gallotannins

Gallic acid, identified by comparison with a commercial standard, was only detected under Experiment 2 conditions, despite the fact its presence in pomegranate juices has been broadly reported [7,13,30]. Gallotannins, composed by monomeric and dimeric galloyl moieties linked to a hexose sugar were also detected. Six compounds matching the molecular ion *m/z* 331, and characteristic fragment ions of monogalloyl-hexoside [5] were observed. In addition, compound **36** revealed a molecular ion at *m/z* 483 and fragments characteristic of digalloyl-hexoside, which was previously reported in pomegranate juice [5]. Concerning the effect of the MS operating mode on the identification of gallic acid-derived phenolics, a better ionisation of glycosidic structures in Experiment 1 was observed. As said, the conditions of Experiment 2 seemed to be better suited for gallic acid without any further conjugation.

#### 2.1.3. Non-Coloured Flavonoids

Fourteen different flavonoids belonging to five subclasses of non-coloured flavonoids (flavan-3-ols, flavonols, flavanones, dihydrochalcones, and flavones) were tentatively identified. The flavan-3-ols detected (compounds **10**, **11**, and **14**) were (+)-catechin, (−)-epicatechin, and (+)-gallocatechin, all previously reported in pomegranate [4]. Experiment 1 allowed the identification of these three flavan-3-ols whilst Experiment 2 source conditions were good for the identification of only (−)-epicatechin.

Flavonol and dihydroflavonol glycosides (compounds **29**, **31**, **33**, **37**, and **44**) were also detected. These compounds have been already described in pomegranate juice [5,21,31]. Compound **30** exhibited a molecular ion at 447 *m/z* and a MS^2^ fragment ion at *m/z* 285, which corresponds to the characteristic loss of *O-*hexoside. MS^3^ fragments were close to those displayed by kaempferol. Nevertheless, the presence of a fragment ion at *m/z* 125, corresponding to the anion of phloroglucinol, was characteristic of 2'-hydroxyflavonols [32]. This fact, together with the shorter retention time of this compound with respect to kaempferol-*O*-hexoside (**29**), prompted us to identify tentatively this compound as datiscetin-*O-*hexoside [33]. To our knowledge, this compound has been described for the first time in pomegranate juice. For method specificity, flavonol derivatives, except datiscetin-*O-*hexoside and dihydrokaempferol-hexoside, were better responsive to the source conditions of Experiment 1 than to those of Experiment 2.

Phlorizin (phloretin-hexoside) (**27**) was identified by comparison with its commercial standard but only under the conditions of Experiment 1. Contrary to this, the aglycone phloretin (**9**) was characterized by its fragment ion spectrum and only detected in Experiment 2. As far as we know, dihydrochalcones have been described in pomegranate juices for the first time in this study. The tentative identification of the flavanone pinocembrin (**6**) was carried out by comparing the obtained fragmentation with previously published data [34]. Another flavanone (compound **8**) shared both molecular ion at *m/z* 271 and fragmentation pattern with a commercial standard of naringenin. However, the retention times did not match, leading us to hypothesise that this compound could be a flavanone similar to naringenin, but with a diverse hydroxylation pattern, likely in the B ring, and could be an intermediate compound in the biosynthesis of pomegranate flavonoids [35]. Compound **8** was observed indistinctly of the experiment used, in contrast to pinocembrin which only occurred within Experiment 2. As in the case dihydrochalcones, flavanones have been reported in pomegranate juice for the first time.

A flavone (compound **7**) was also tentatively identified according to the typical fragmentation pattern of tryhydroxyflavones, such as baicalein and norwogonin, as described by Wang *et al*. [36]. To our knowledge, the presence of flavones in pomegranate juices had not been previously reported.

#### 2.1.4. Phenolic Acid Derivatives

A total of five phenolic acid derivatives were found in pomegranate juice in this study. Compounds derived from hydroxybenzoic acids were predominant. Compounds **2**, **15**, **4**, and **12** were identified as vanillic acid, its hexoside, syringaldehyde, and hydroxybenzoic acid hexoside, respectively, by comparison of their mass fragment profiles with previously published data [5,37,38]. To our knowledge, all these substances, except vanillic acid hexoside, have been reported in pomegranate juice for the first time in this study. Moreover, a hydroxycinnamic acid derivative (compound **25**) was tentatively identified as coumaric acid for its typical MS^2^ fragments (*m/z* 163 and 119) [5].

**Figure 3:**
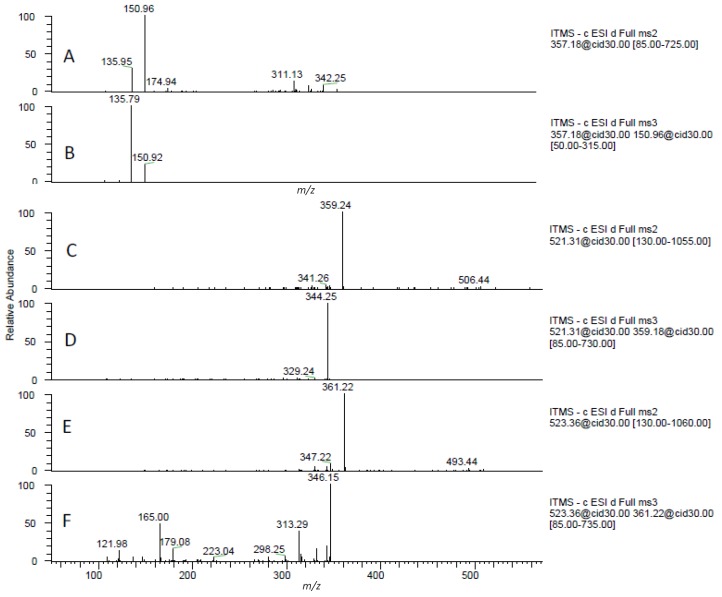
MS^2^ and MS^3^ fragment ion spectra of pinoresinol (**A** and **B**), cycolariciresinol hexoside (**C** and **D**) and secoisolariciresinol hexoside (**E** and **F**).

In this case, phenolic acid derivatives with sugar moieties were better identified by experiment 1, whereas Experiment 2 was more appropriate for aglycones, indistinctly of the substitution pattern (hydroxyl and methyls).

#### 2.1.5. Lignans

A total of six lignans, phenylpropanoidic secondary metabolites with estrogenic activity, were also identified in the pomegranate juice by comparison with published data (Figure 3) [39,40]. The aglycones of pinoresinol (**22**), secoisolariciresinol (**23**), and cyclolariciresinol had been previously reported in pomegranate products [41,42]. Compounds **38**–**41** have never been described in pomegranate juice. In addition, this is the first time that glycosylated lignans (compounds **39**–**41**) have been identified in pomegranate juices. A possible explanation behind the presence of these lignans glycosides could be the lack of hydrolysis procedures prior to MS analysis. This procedure, described in other works, may alter the structure of lignans and, hence, provide misleading results [41,42]. In the present work samples were directly analyzed without previous preparation.

#### 2.1.6. Organic Acids

In the first 2 min of the chromatographic analysis, organic acids were separated and identified by comparison with published data [12,43]. Citric acid and L-malic acid have been pointed out as the main organic acids in pomegranate juices and are considered valuable markers to group cultivars [44]. These compounds (**1**, **5**, and **24**) were not within the aim of the present work, but some interesting points should be considered. Experiment 1 allowed the identification of these three compounds whereas Experiment 2 only was sensitive to the sole citric acid. Differences could be attributed to the number of free carboxylic acid: two in the case of L-malic and likely the citric acid derivative, and three for citric acid. This information could be useful in order to understand how different operating conditions may affect the identification of phytochemicals sharing similar chemical substitutions. Furthermore, the identification of these organic acids together with (poly)phenols might lead to an integrated method for fingerprinting of phytochemicals in pomegranate juice and other vegetal matrices as well as to elucidate their different ratios during fruit ripening.

#### 2.1.7. Anthocyanin Identification by Using Positive Mode

Analyses performed in positive mode resulted in the identification of six anthocyanins and a flavanol-anthocyanin adduct. The identities of the compounds as well as their mass spectral characteristics are summarized in Table 2. Anthocyanins are the phenolics responsible for the red colour of pomegranate juice. The anthocyanin profile comprised cyanidin, pelargonidin, and delphinidin, all conjugated with one or two hexose sugars (compounds **POS1**–**6**). These sugar moieties could safely be considered 3-*O-*glucosides and 3,5-diglucosides, according to the typical fingerprint of pomegranate juice authenticity [6,11]. Elution order of anthocyanins was in agreement with the polarity of their respective anthocyanidins and with the number of attached sugar molecules. In addition, classical fragmentation patterns of anthocyanins in ESI positive experiments were recorded and the sequential loss of individual sugars at every induced collision was observed [5]. Moreover, anthocyanins were unambiguously identified thanks to analyses performed in negative mode where fragmentation patterns matched with those reported by Sun *et al*. [45] (data not shown).

**Table 2:** Identification of phytochemical compounds by UHPLC-MS^n^ in positive mode by different analytical columns.

Id.	Compounds	[M]^+^ (*m/z*)	MS^2^ ion fragments (*m/z*) *^a^*	MS^3^ ion fragments (*m/z*) *^a^*	BlueOrchid C18		Hypersil Gold C18		Kinetex PFP	
					RT (min)		RT (min)		RT (min)	
POS1	Pelargonidin-3-glucoside	**433**	**271** *^b^*		6,40	x	6,12	x	6,22	x
POS2	Cyanidin-3-glucoside	**449**	**287**		6,08	x	5,80	x	5,82	x
POS3	Delphinidin-3-glucoside	**465**	**303**		5,80	x	5,52	x	5,50	x
POS4	Pelargonidin-3,5-diglucoside	**595**	**433**, 271	271	5,70	x	5,47	x	5,38	x
POS5	Cyanidin-3,5-diglucoside	**611**	**449**, 287	287	5,52	x	5,16	x	5,17	x
POS6	Delphinidin-3,5-diglucoside	**627**	**465**, 303	303	5,28	x	5,00	x	4,88	x
POS7	(epi)gallocatechin-cyanidin-3-hexoside	**753**	**591**	423, 573, 329, 465, 287	5,13	x				

*^a^* Fragment ions are listed in order of relative abundances; *^b^* MS^2^ ions in bold were those subjected to MS^3^ fragmentation.

An adduct formed by the direct condensation between a flavanol and an anthocyanin was also found (**POS7**). The adduct (epi)gallocatechin-cyanidin-3-hexoside, with a molecular ion *m/z* 753, was identified according to its fragmentation pathway and the series of characteristic ions previously described [46]. This kind of adduct has been pointed out to be present at very small concentrations in pomegranate juices [46].

#### 2.1.8. General Discussion about the Use of This New Approach for Phytochemical Identification

**Figure 4:**
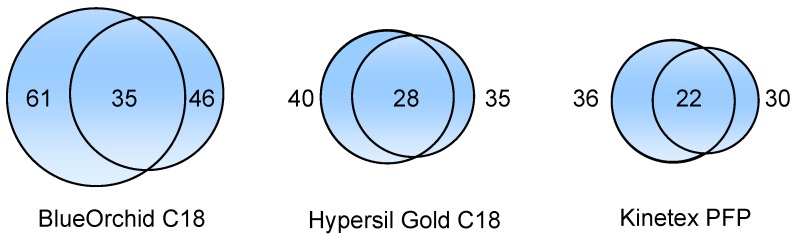
Venn diagrams showing the number of compounds identified in each column by using Experiment 1 conditions (on the left) and Experiment 2 conditions (on the right) as well as the number of compounds elucidated by both negative mode conditions (in the center).

The described approach, combining three different MS operating conditions, allowed the tentative identification of a total of 75 compounds in pomegranate juice. The use of the two experiments in negative mode with different ionization and fragmentation conditions allowed the tentative identification of up to 68 compounds. Furthermore, MS operating methods played a major role on the identification of phytochemicals by data-dependent experiment outcomes. In Figure 4, Venn diagrams show the number of compounds identified by using different experiments in negative mode (Experiments 1 and 2) as well as the number of compounds elucidated by both experimental conditions. Overall, indistinctly of the chromatographic column used, Experiment 1 allowed the tentative identification of a wider number of phytochemicals with respect to Experiment 2. Nonetheless, the use of the two operating modes favoured a better understanding of the phytochemical fingerprint of the analysed pomegranate juice.

Experiment 1 conditions were developed by optimizing the MS tune for epicatechin analysis. In fact, the better performance was for the identification of flavonoidic structures. Moreover, these conditions also showed a particular selectivity for low molecular weight lignans and gallotannins. On the contrary, Experiment 2 conditions, developed by the optimization of MS for punicalagin detection, allowed a better identification of high molecular weight ellagitannins. On the basis of these results, Experiment 1 could be regarded as a preliminary polyphenol investigation, whereas Experiment 2 could be specially considered for the elucidation of complex and heavy ellagitannins.

The phytochemical composition of pomegranate has been extensively studied, with some important and detailed recent works [5,11,12,13,14,21]. This has highlighted the broad array of phenolic structures present in pomegranate juices. However, all these works have been performed with a specific focus on certain groups of pomegranate phytochemicals [12,13,42], and the applied methodologies consisted of complex and long sample preparations and time-consuming chromatographic conditions [5,11,21]. In contrast, our work is characterized by a quick, high throughput screening of its complete profile, with chromatographic analyses performed in less than one hour altogether. Moreover, this work allowed the tentative identification of 21 compounds not previously reported in this well-known vegetal matrix.

### 2.2. Comparison of Pomegranate Juice LC/MS Based Fingerprinting among Different Chromatographic Columns

The best analytical conditions for rapid UHPLC-MS^n^ evaluation of pomegranate juice phytochemicals were assessed by comparing three different chromatographic columns. UHPLC allows the use of columns with small particle size, which can speed up the chromatographic analyses. C18 columns are those most often used [17] for the separation of these classes of compounds. For this reason, two different C18 columns (BlueOrchid C18 and Hypersil Gold C18) were tested along with a pentafluorophenyl (PFP) column (Kinetex PFP). Perfluorinated columns have exhibited good resolution for the study of phenolic compounds, just as a different selectivity from C18 columns [47].

The paramount role of the analytical column on the pomegranate juice profiling was evident based on the number of compounds that were successfully identified using different columns. The separation performed with BlueOrchid C18 resulted in a total of 75 identified peaks. Hypersil Gold C18 and Kinetex PFP were associated to the identification of 50 and 48 phytochemicals, respectively.

As different MS operating conditions were used, comparison between them should be taken into account. In the case of the positive ionization applied for anthocyanin identification, the three columns rendered a classical profile, comprising six anthocyanins [6]. Nonetheless, the use the BlueOrchid C18 allowed the separation and consequent identification of the flavanol-anthocyanin adduct (**POS7**) which was not detected with the other two columns (Table 2).

**Figure 5:**
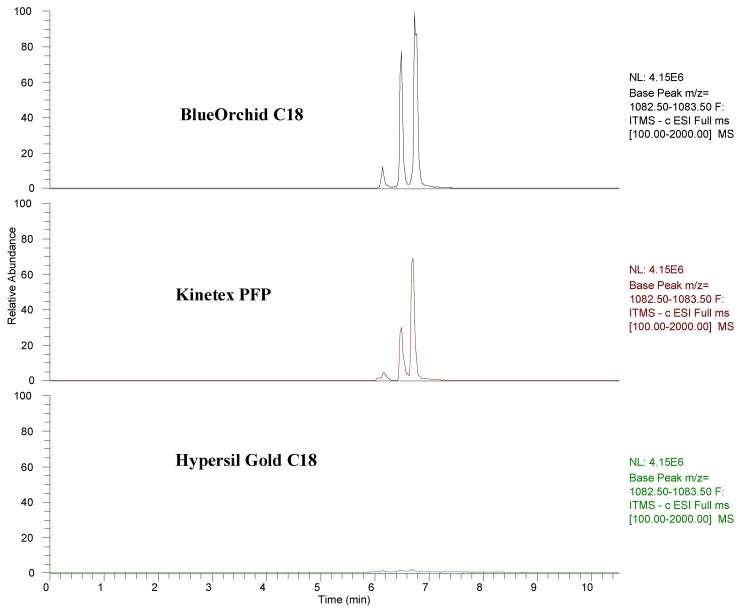
Chromatograms of punicalagins in full MS Scan of the three analytical columns.

Relating to the two experiments in negative mode, broad differences among columns were observed (Figure 4). Despite the aforementioned notably differences among experiments, data pointed out how BlueOrchid C18 was always the best column by number of identified phytochemicals, followed by Hypersil Gold C18 and Kinetex PFP. In accordance with these results, C18 columns could be regarded as the best solution for analysis of pomegranate juices. However, based on the effectiveness reached in this set of experiments, the selection of a specific C18 column could be considered extremely relevant as they can display distinct performances [48]. This is also confirmed by the fact that Hypersil Gold C18 was not able to efficiently elute punicalagin isomers, contrary to BlueOrchid C18 (Figure 5).

PFP column did not allow the identification of lignans and the ellagitannin with *m/z* 1101, identified as punicalagin-like by Borges *et al*. [11]. This fact should be taken into consideration, as punicalagin-like has been confirmed as the main ellagitannin of pomegranate juices [49] and lignans could be promising phytoestrogens [42]. In general, even if the reasons are still unclear, the phytochemical profile of pomegranate juices might be underestimated if analyses are performed with a Kinetex PFP.

## 3. Experimental

### 3.1. Pomegranate Juice and Sample Preparation

Pomegranate fruits from cv. *Mollar de Elche* were freshly collected from a commercial farm in the Murcia region (SE Spain). Pomegranates were cut in halves and the juice was obtained by pressure with a laboratory press (Zumonat C-40; Somatic AMD, Valencia, Spain) [50]. Juice was stored frozen (−20 °C) until analysis. Suitable aliquots of pomegranate juice were centrifuged for 5 min at 14,000 rpm at room temperature. Then, the supernatant was filtered through a 0.45 μm nylon membrane before UHPLC-MS^n^ analysis, without further processing.

### 3.2. UHPLC-MS^n^ Analyses

The juices were analyzed using an Accela UHPLC 1250 equipped with a linear ion trap-mass spectrometer (LTQ XL, Thermo Fisher Scientific Inc., San Jose, CA, USA) fitted with a heated-electrospray ionization probe (H-ESI-II; Thermo Fisher Scientific Inc). Separations were performed using three different columns: (*i*) BlueOrchid C18 (50 × 2 mm), 1.8 µm particle size (Knauer, Berlin, Germany); (*ii*) Hypersil Gold C18 (50 × 2.1 mm), 1.9 µm particle size (Thermo Fisher Scientific Inc); (*iii*) Kinetex PFP (50 × 2.1 mm), 2.6 µm particle size (Phenomenex, Macclesfield, UK). Volume injected was 5 µL and column oven was set to 30 °C.

Three MS experiments were performed for each analytical column, of which two in negative mode and one using positive ionization (anthocyanins). In negative mode, a preliminary polyphenol investigation was carried out using the following conditions, optimized for epicatechin analysis (Experiment 1). The MS worked with a capillary temperature equal to 275 °C, while the source heather temperature was set to 200 °C. The sheath gas flow was 40 units, while both auxiliary and sweep gas were set to 5 units. The source voltage was 4 kV. The capillary voltage and tube lens were −42 and −118 V, respectively. The mobile phase, pumped at a flow rate of 0.2 mL/min, was kept at 0.1% aqueous formic acid up to 2 min, and then 3-min linear gradient of 0% to 20% acetonitrile in 0.1% formic acid. From 5 to 13 min the acidified acetonitrile turned up to 40%, followed to 3 min of 80% acetonitrile and then 4 min at the start conditions to reequilibrate the column. Analyses were carried out using full scan, data-dependent MS^3^ scanning from *m/z* 100 to 2,000, with collision induced dissociation (CID) equal to 30 (arbitrary units).

Then, the MS was optimized to hydrolyzable tannin analysis, after infusion with punicalagin standard (mixture of two isomers) (Experiment 2). The capillary temperature was set to 275 °C, while the source heather temperature was 250 °C. The sheath gas flow was 60 units, while auxiliary and sweep gas were set to 15 and 4 units, respectively. The source voltage was 4 kV. The capillary voltage and tube lens were −49 and −153 V, respectively. Analyses were carried out using full scan, data-dependent MS^3^ scanning from *m/z* 100 to 2,000, with CID equal to 13.8 in MS^2^ and 30 in MS^3^. The function “Stepped Collision Energy” using a width of 20% and three collision energy steps was activated. The chromatographic conditions were the same used for preliminary phenolic analyses.

In positive mode, the MS worked with a capillary temperature equal to 275 °C, while the source heather temperature was set to 300 °C. The sheath gas flow was 40 units, while auxiliary gas was set to 5 units, without sweep gas. The source voltage was 4.5 kV. The capillary voltage and tube lens were 20 and 95 V, respectively. The mobile phase, pumped at a flow rate of 0.2 mL/min, was a 12-min linear gradient of 5% to 30% acetonitrile in 0.1% aqueous formic acid, followed to 2 min of 80% acetonitrile and then 3 min at the start conditions to re-equilibrate the column. Analyses were carried out using full scan, data-dependent MS^2^ and MS^3^ scanning from *m/z* 50 to 1,000, with collision induced dissociation (CID) equal to 15 (arbitrary units). The function “Stepped Collision Energy” using a width of 20% and 3 collision energy steps was also activated. Pure helium gas was used for CID.

The activation time used was 60 milliseconds and one precursor ion (the most intense) was used for MS^3^ scanning. The elapsed scan time for experimental condition 1 was 0.19 s, while for experimental 2 and anthocyanins (positive ionization) was 0.38 s. MS^2^ and MS^3^ data dependent outputs ranged from 150 to 400, depending on the chromatographic column and experimental condition used.

## 4. Conclusions

The best chromatographic conditions for UHPLC separation as well as a new procedure for non-targeted screening of (poly)phenolic compounds in pomegranate juice were assessed. The use of three different MS operating conditions resulted in a quick and high throughput screening of a complete pomegranate profile. Furthermore, this comprehensive evaluation allowed the tentative identification of 21 phytochemicals not previously reported in pomegranate juice, extending, hence, the variety of substances contributing to the definition and to the bioactive properties of this plant food. Likewise, the importance of an appropriate analytical column selection was pinpointed.

In terms of applied research, the method allows to meet different needs, as its enforcement may be suggested for primary routine screening of large number of production batches in industrial production or for quality control in authenticity/adulteration detection, an issue whose relevance is increasing in the marketplace. As it can guarantee comprehensive information with limited effort, the high-throughput potential of this method could assist the phytochemical screening of different vegetal matrices. In addition, this approach could serve to improve data contained in food composition tables, but further work should be performed in order to evaluate the role of the source ionization conditions in the subsequent identification of phytochemicals in vegetal matrices.

## References

[1] Gasperotti M., Masuero D., Vrhovsek U., Guella G., Mattivi F. (2010). Profiling and accurate quantification of *Rubus* ellagitannins and ellagic acid conjugates using direct UPLC-Q-TOF hdms and HPLC-DAD analysis. J. Agric. Food Chem..

[2] Mulabagal V., Calderón A.I. (2012). Liquid chromatography/mass spectrometry based fingerprinting analysis and mass profiling of *Euterpe oleracea* (açaí) dietary supplement raw materials. Food Chem..

[3] Mena P., Gironés-Vilaplana A., Moreno D.A., García-Viguera C. (2011). “Pomegranate fruit for health promotion: Myths and realities” In Antioxidant Properties of Crops III. Funct. Plant Sci. Biotechnol..

[4] De Pascual-Teresa S., Santos-Buelga C., Rivas-Gonzalo J.G. (2000). Quantitative analysis of flavan-3-ols in Spanish foodstuffs and beverages. J. Agric. Food Chem..

[5] Fischer U.A., Carle R., Kammerer D.R. (2011). Identification and quantification of phenolic compounds from pomegranate (*Punica granatum* L.) peel, mesocarp, aril and differently produced juices by HPLC-DAD-ESI/MS^n^. Food Chem..

[6] Gil M.I., García-Viguera C., Artés F., Tomás-Barberán F.A. (1995). Changes in pomegranate juice pigmentation during ripening. J. Sci. Food Agric..

[7] Gil M.I., Tomás-Barberán F.A., Hess-Pierce B., Holcroft D.M., Kader A.A. (2000). Antioxidant activity of pomegranate juice and its relationship with phenolic composition and processing. J. Agric. Food Chem..

[8] Ben Nasr C., Ayed N., Metche M. (1996). Quantitative determination of the polyphenolic content of pomegranate peel. Z. Lebensm. Unters. Forsch..

[9] Zhang Y., Krueger D., Durst R., Lee R., Wang D., Seeram N., Heber D. (2009). International multidimensional authenticity specification (IMAS) algorithm for detection of commercial pomegranate juice adulteration. J. Agric. Food Chem..

[10] Plumb G.W., de Pascual-Teresa S., Santos-Buelga C., Rivas-Gonzalo J.C., Williamson G. (2002). Antioxidant properties of gallocatechin and prodelphinidins from pomegranate peel. Redox Rep..

[11] Borges G., Mullen W., Crozier A. (2010). Comparison of the polyphenolic composition and antioxidant activity of European commercial fruit juices. Food Funct..

[12] Cristofori V., Caruso D., Latini G., Dell’Agli M., Cammilli C., Rugini E., Bignami C., Muleo R.  (2010). Fruit quality of Italian pomegranate (*Punica granatum* L.) autochthonous varieties. Eur. Food Res. Technol..

[13] Romani A., Campo M., Pinelli P. (2012). HPLC/DAD/ESI-MS analyses and anti-radical activity of hydrolyzable tannins from different vegetal species. Food Chem..

[14] Borges G., Crozier A. (2012). HPLC-PDA-MS fingerprinting to assess the authenticity of pomegranate beverages. Food Chem..

[15] Törrönen A.R., Quideau S. (2009). Sources and health effects of dietary ellagitannins. Chemistry and Biology of Ellagitannins.

[16] Filigenzi M.S., Ehrke N., Aston L.S., Poppenga R.H. (2011). Evaluation of a rapid screening method for chemical contaminants of concern in four food-related matrices using QuEChERS extraction, UHPLC and high resolution mass spectrometry. Food Addit. Contam. A.

[17] Di Stefano V., Avellone G., Bongiorno D., Cunsolo V., Muccilli V., Sforza S., Dossena A., Drahos L., Vékey K. (1259). Applications of liquid chromatography-mass spectrometry for food analysis. J. Chromatogr. A.

[18] Rak G., Fodor P., Abrankó L. (2010). Three-step HPLC-ESI-MS/MS procedure for screening and identifying non-target flavonoid derivatives. Int. J. Mass Spectrom..

[19] Del Rio D., Rodríguez-Mateos A., Spencer J.P., Tognolini M., Borges G., Crozier A.  (2012). Dietary (Poly)phenolics in Human Health: Structures, Bioavailability, and Evidence of Protective Effects Against Chronic Diseases. Antioxid. Redox Signal..

[20] Hukkanen A.T., Kokko H.I., Buchala A.J., McDougall G.J., Stewart D., Kärenlampi S.O., Karjalainen R.O. (2007). Benzothiadiazole induces the accumulation of phenolics and improves resistance to powdery mildew in strawberries. J. Agric. Food Chem..

[21] Fischer U.A., Dettmann J.S., Carle R., Kammerer D.R. (2011). Impact of processing and storage on the phenolic profiles and contents of pomegranate (*Punica granatum* L.) juices. Eur. Food Res. Technol..

[22] Kähkönen M., Kylli P., Ollilainen V., Salminen J.P., Heinonen M. (2012). Antioxidant activity of isolated ellagitannins from red raspberries and cloudberries. J. Agric. Food Chem..

[23] Barry K.M., Pearce R.B., Mohammed C.M. (2000). Properties of reaction zones associated with decay from pruning wounds in plantation-grown *Eucalyptus nitens*. For. Pathol..

[24] De Rijke E., Zappey H., Ariese F., Gooijer C., Brinkman U.A.T. (2003). Liquid chromatography with atmospheric pressure chemical ionization and electrospray ionization mass spectrometry of flavonoids with triple-quadrupole and ion-trap instruments. J. Chromatogr. A.

[25] Arnao M.B., Cano A., Alcolea J.F., Acosta M. (2001). Identification of hydrolysable tannins in the reaction zone of Eucalyptus nitens wood by high performance liquid chromatography-electrospray ionisation mass spectrometry. Phytochem. Anal..

[26] Boulekbache-Makhlouf L., Meudec E., Chibane M., Mazauric J.P., Slimani S., Henry M., Cheynier V., Madani K. (2010). Analysis by high-performance liquid chromatography diode array detection mass spectrometry of phenolic compounds in fruit of *Eucalyptus globulus* cultivated in Algeria. J. Agric. Food Chem..

[27] Venzie J.L., Castro J., Balarama Krishna M.V., Nelson D.M., Marcus R.K. (2007). Electron-impact and glow-discharge ionization LC-MS analysis of green tea tincture. Anal. Bioanal. Chem..

[28] Zywicki B., Reemtsma T., Jekel M. (2002). Analysis of commercial vegetable tanning agents by reversed-phase liquid chromatography-electrospray ionization-tandem mass spectrometry and its application to wastewater. J. Chromatogr. A.

[29] Vrhovsek U., Guella G., Gasperotti M., Pojer E., Zancato M., Mattivi F. (2012). Clarifying the identity of the main ellagitannin in the fruit of the strawberry, *Fragaria vesca* and *Fragaria ananassa* Duch. J. Agric. Food Chem..

[30] Mena P., Gironés-Vilaplana A., Martí N., García-Viguera C. (2012). Pomegranate varietal wines: Phytochemical composition and quality parameters. Food Chem..

[31] He L., Xu H., Liu X., He W., Yuan F., Hou Z., Gao Y.  (2011). Identification of phenolic compounds from pomegranate (*Punica granatum* L.) seed residues and investigation into their antioxidant capacities by HPLC-ABTS+ assay. Food Res. Int..

[32] McNab H., Ferreira E.S.B., Hulme A.N., Quye A. (2009). Negative ion ESI-MS analysis of natural yellow dye flavonoids-An isotopic labelling study. Int. J. Mass Spectrom..

[33] Petroviciu I., Vanden Berghe I., Cretu I., Albu F., Medvedovici A. (2012). Identification of natural dyes in historical textiles from Romanian collections by LC-DAD and LC-MS (single stage and tandem MS). J. Cult. Herit..

[34] Liu R., Ye M., Guo H., Bi K., Guo D.A. (2005). Liquid chromatography/electrospray ionization mass spectrometry for the characterization of twenty-three flavonoids in the extract of *Dalbergia odorifera*. Rapid Commun. Mass Spectrum..

[35] Li H., Qiu J., Chen F., Lv X., Fu C., Zhao D., Hua X., Zhao Q. (2012). Molecular characterization and expression analysis of dihydroflavonol 4-reductase (DFR) gene in *Saussurea medusa*. Mol. Biol. Rep..

[36] Wang Y., Yang L., He Y.Q., Wang C.H., Welbeck E.W., Bligh S.W.A., Wang Z.T. (2008). Characterization of fifty-one flavonoids in a Chinese herbal prescription Longdan Xiegan Decoction by high-performance liquid chromatography coupled to electrospray ionization tandem mass spectrometry and photodiode array detection. Rapid Commun. Mass Spectrum..

[37] Perestrelo R., Lu Y., Santos S.A.O., Silvestre A.J.D., Neto C.P., Câmara J.S., Rocha S.M.  (2012). Phenolic profile of Sercial and Tinta Negra *Vitis vinifera* L. grape skins by HPLC-DAD-ESI-MSn: Novel phenolic compounds in* Vitis vinifera* L. grape. Food Chem..

[38] Sanz M., Fernández de Simón B., Esteruelas E., Muñoz A.M., Cadahía E., Hernández T., Estrella I., Pinto E. (2011). Effect of Toasting Intensity at Cooperage on Phenolic Compounds in Acacia (*Robinia pseudoacacia*) Heartwood. J. Agric. Food Chem..

[39] Eklund P.C., Backman M.J., Kronberg L.Å., Smeds A.I., Sjöholm R.E.  (2008). Identification of lignans by liquid chromatography-electrospray ionization ion-trap mass spectrometry. J. Mass Spectrom..

[40] Huis R., Morreel K., Fliniaux O., Lucau-Danila A., Fénart S., Grec S., Neutelings G., Chabbert B., Mesnard F., Boerjan W. (2012). Natural hypolignification is associated with extensive oligolignol accumulation in flax stems. Plant Physiol..

[41] Bonzanini F., Bruni R., Palla G., Serlataite N., Caligiani A.  (2009). Identification and distribution of lignans in *Punica granatum* L. fruit endocarp, pulp, seeds, wood knots and commercial juices by GC-MS. Food Chem..

[42] Fischer U.A., Jaksch A.V., Carle R., Kammerer D.R. (2012). Determination of lignans in edible and nonedible parts of pomegranate (*Punica granatum* L.) and products derived therefrom, particularly focusing on the quantitation of isolariciresinol using HPLC-DAD-ESI/MS^n^. J. Agric. Food Chem..

[43] Sawada Y., Akiyama K., Sakata A., Kuwahara A., Otsuki H., Sakurai T., Saito K., Hirai M.Y. (2009). Widely targeted metabolomics based on large-scale MS/MS data for elucidating metabolite accumulation patterns in plants. Plant Cell Physiol..

[44] Mena P., García-Viguera C., Navarro-Rico J., Moreno D.A., Bartual J., Saura D., Martí N.  (2011). Phytochemical characterisation for industrial use of pomegranate (*Punica granatum* L.) cultivars grown in Spain. J. Sci. Food Agric..

[45] Sun J., Lin L.Z., Chen P. (2012). Study of the mass spectrometric behaviors of anthocyanins in negative ionization mode and its applications for characterization of anthocyanins and non-anthocyanin polyphenols. Rapid Commun. Mass Spectrum..

[46] Sentandreu E., Navarro J.L., Sendra J.M.  (2010). LC-DAD-ESI/MS^n^ determination of direct condensation flavanol-anthocyanin adducts in pressure extracted Pomegranate (*Punica granatum* L.) juice. J. Agric. Food Chem..

[47] Regos I., Treutter D. (2010). Optimization of a high-performance liquid chromatography method for the analysis of complex polyphenol mixtures and application for sainfoin extracts (*Onobrychis viciifolia*). J. Chromatogr. A.

[48] Haghedooren E., Janssens T., Nijs R., Park S.K., Farkas E., Dragovic S., Noszál B., Hoogmartens J., Adams E. (2009). Selecting a suitable LC column for pharmaceutical separations using a column characterisation system. J. Liq. Chromatogr. Relat. Technol..

[49] Mena P., Martí N., Saura D., Valero M., García-Viguera C. (2012). Combinatory Effect of Thermal Treatment and Blending on the Quality of Pomegranate Juices. Food Bioprocess Technol..

[50] Pérez-Vicente A., Serrano P., Abellán P., García-Viguera C. (2004). Influence of packaging material on pomegranate juice colour and bioactive compounds, during storage. J. Sci. Food Agric..

